# Molecular Origin of Aneotropy and Related Surface Tension Anomalies in Hydrogenated and Fluorinated Alcohol Mixtures: New Experimental Data and Theoretical Molecular Modeling

**DOI:** 10.1021/acs.langmuir.6c00218

**Published:** 2026-04-09

**Authors:** João Duarte, Diogo Machacaz, Teresa Pires, Tiago M. Eusébio, Pedro Morgado, Lourdes F. Vega, Eduardo J. M. Filipe

**Affiliations:** † Centro de Química Estrutural, Institute of Molecular Sciences, Instituto Superior Técnico, 426092 Universidade de Lisboa, 1049-001 Lisboa, Portugal; ‡ Research and Innovation Center on CO_2_ and Hydrogen (RICH Center), 105955 Khalifa University of Science and Technology, P.O. Box 127788, Abu Dhabi, United Arab Emirates; § Department of Chemical and Petroleum Engineering, Khalifa University of Science and Technology, P.O. Box 127788, Abu Dhabi, United Arab Emirates

## Abstract

Aneotropy is an intriguing interfacial phenomenon that is still poorly understood and displayed by several relevant mixtures. This work is a contribution toward the molecular-level interpretation of aneotropy and related surface tension anomalies, combining new experimental data, theoretical calculations, and atomistic molecular dynamics (MD) simulations, applied to carefully chosen systems. First, bulk and interfacial properties of the hexane/perfluorohexane mixture were modeled with the soft-SAFT and soft-SAFT-DGT approaches, respectively. This mixture, governed exclusively by dispersion forces, is well-known by the mutual phobicity between hydrogenated and perfluorinated chains. The theory captures the very positive excess volumes and enthalpies and predicts the negative aneotrope, revealing that the weak interactions between unlike molecules, which are responsible for the large deviations to ideality in the bulk liquid, are even more unfavorable at the interface, leading to large surface anomalies. The effect of introducing an associative OH group into molecules of an equivalent chain length was then addressed. Three mixtures of hydrogenated and fluorinated alcohols were studied, extending to longer chains a previous work involving mixtures of short chain alcohols. New experimental data were measured for the surface tension of butanol/1*H*,1*H*-perfluorobutanol, hexanol/1*H*,1*H*-perfluorohexanol, and decanol/1*H*,1*H*-perfluorooctanol mixtures at 298.2K. The bulk properties of the mixtures were modeled with MD simulations and soft-SAFT, relying on the description of the dispersive interactions previously determined, and the interfacial behavior was predicted with soft-SAFT-DGT. Although these components interact strongly through hydrogen bonding, the surface tension of the mixtures displays very large negative deviations from the weighted average and in some cases shallow negative aneotropes, shallower than that displayed by the hexane/perfluorohexane mixture. The theoretical predictions reproduce the experimental data and provide a molecular-level interpretation of these unusual interfacial anomalies, advancing our fundamental understanding of complex fluid interfaces.

## Graphical abstract

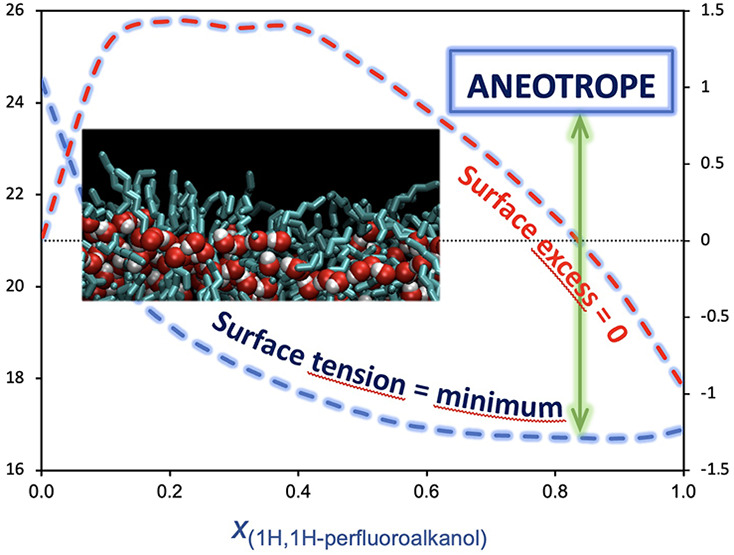

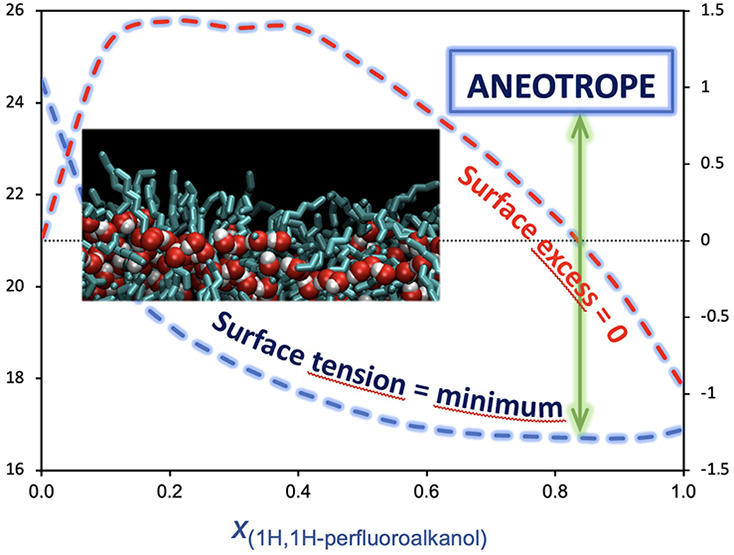

## Introduction

Aneotropy was first observed and named by Ian McLure^1^ to designate the existence of minima (or in even rarer cases maxima) in the surface tension versus composition isotherms of mixtures. This is an unusual and still poorly understood phenomenon. At a negative aneotrope, the surface tension of mixtures reaches values lower than that of the less tense component, a singular behavior that cannot be exclusively explained by adsorption of the less tense component at the interface. The existence of negative aneotropy indicates a reduction of the cohesiveness of the bulk or surface layer resulting from unfavorable molecular interactions.

Negative aneotropy is generally accompanied by very large deviations from the weighted average surface tension of the pure components and by horizontal inflections, which in many cases obscure the aneotropes. This diversity of anomalies affecting the interfacial behavior of mixtures has greatly contributed to the poor global understanding of the phenomenon.

In a subsequent series of papers,^2–5^ McLure and co-workers tried a systematic description of the subject and theoretical modeling using the regular solution theory. Four main types of surface tension isotherms were identified, and the existence of anomalies was associated with the proximity of the mixture critical points. However, Fouad and Vega^6^ have shown that azeotropy and the proximity of an UCST are not necessary conditions for aneotropy and horizontal inflections.

In most of the known systems displaying negative aneotropes, the unfavorable interactions are also expressed in the liquid phase as positive deviations to Raoult’s law, positive azeotropy, and even liquid–liquid immiscibility. These involve mixtures of hydrogenated and perfluorinated substances, whose behavior is determined by the mutual phobicity between the two types of segments.^1–5,7–9^ Aneotropes have also been observed in binary mixtures of ethanol with alkanes, octane, decane, dodecane, and tetradecane.^10–12^ In these cases, the similarity of the surface tensions of the two components contributes to the aneotrope.

In a recent publication,^13^ we reported new experimental data for the surface tension of mixtures of 2,2,2-trifluoroethanol (TFE) with three hydrogenated alcohols (ethanol, propanol, and butanol). All mixtures display well-defined negative aneotropes that, as the chain length of the hydrogenated alcohol increases, become less pronounced and progressively deviate toward compositions closer to the fluorinated component. It should be realized, however, that unlike the previously mentioned examples,^1–4,7–12^ these TFE/alkanol mixtures do not interact weakly, as they associate through hydrogen bonding and display negative deviations from Raoult’s law (and even negative excess enthalpy in the case of the TFE/ethanol mixture).^14^ At the surface, the negative aneotropy indicates that the weak interactions between fluorinated and hydrogenated chains become dominant, probably due to orientational constraints.

Hydrogenated and perfluorinated chains are well-known to be mutually phobic, which leads to positive deviations to Raoult’s law, large positive excess properties, and liquid–liquid immiscibility.^15–17^ Nanodomains have been detected in these mixtures,^18,19^ as well as anomalies in their transport^20,21^ and conformational properties.^14,22^ Several examples demonstrate the large impact of the phobicity between hydrogenated and perfluorinated chains on the organization of supramolecular structures.^23–27^

Mixtures of hydrogenated and fluorinated alcohols display a very complex behavior in the liquid^14,28–32^ and gaseous phases.^33–35^ Liquid alcohols, both hydrogenated and perfluorinated, form hydrogen bonds sequentially, creating a dynamic hydrophilic network.^36^ The alkyl chains occupy the space between the polar domains and, in the case of mixtures of hydrogenated and fluorinated alcohols, additionally try to avoid each other. With or without water, the bulk liquid displays a complex microheterogeneous organization featuring polar/aqueous, hydrogenated, and fluorinated domains,^29^ the result of a balance between maximizing hydrogen bonding and the weak London forces between the hydrogenated and perfluorinated chains.

An important motivation to study the interfacial behavior of perfluorinated substances stems from their environmental impact. In addition to advancing the fundamental understanding of these systems, knowing the interfacial behavior of perfluorinated substances is needed from an environmental perspective. Owing to their exceptional chemical stability, the accumulation of these compounds in the environment has become a major concern, fostering the urgent development of remediation strategies, which frequently involve interfacial processes, such as adsorption at solid–liquid and liquid–vapor interfaces. With that in mind, we have been systematically measuring the surface tension^37^ and diffusion coefficients of fluorinated alcohols,^38–40^ as well as other thermophysical properties,^41–43^ which are essential to the optimization of those processes.

In this work, we start by modeling the bulk excess properties of the hexane/perfluorohexane mixture, using data from the literature, with the molecular-based soft-SAFT equation of state^44^ to develop and validate the molecular model and parameters. Soft-SAFT coupled with density gradient theory (DGT)^45^ is then used to predict the interfacial behavior of the same system. Since in this mixture the two components interact exclusively through dispersion forces, this procedure quantifies their contribution to the excess properties and the interfacial behavior, including the existence of the aneotrope. The mutual phobicity between hydrogenated and perfluorinated chains is thus intrinsically incorporated into the model. Additionally, we report new experimental data for the surface tension of three mixtures of fluorinated and hydrogenated alcohols, namely, butanol/1*H*,1*H*-perfluorobutanol, hexanol/1*H*,1*H*-perfluorohexanol, and decanol/1*H*,1*H*-perfluorooctanol, at 298.2 K. This extends our previous study involving short alcohols to molecules with significantly larger chain lengths, enhancing the contribution of dispersion forces and thus the phobicity between the hydrogenated and perfluorinated chains, while keeping the impact of hydrogen bonding between the two alcohols. To the best of our knowledge, this is the first study addressing the surface behavior of these mixtures. The bulk properties were modeled with molecular dynamics (MD) simulations and soft-SAFT, allowing the optimization of soft-SAFT parameters for the association, while keeping the description of the dispersive interactions previously incorporated into the theory. The interfacial properties were then modeled with soft-SAFT-DGT. This strategy enables separating the contributions of the dispersive and associative forces to both the bulk and interfacial properties. Excellent agreement was found between the theoretical predictions and the experimental results, validating the physical models and parametrization and providing molecular-level insight into the interfacial anomalies displayed by these mixtures.

## Experimental Section

### Materials

All alcohols, 1*H*,1*H*-perfluorobutanol (Apollo Scientific, CAS Registry No. 375-01-9), 1*H*,1*H*-perfluorohexanol (Apollo Scientific, CAS Registry No. 423-46-1), 1*H*,1*H*-perfluorooctanol (Sigma-Aldrich, 98%, CAS Registry No. 307-30-2), butanol (Sigma-Aldrich, 99.8%, CAS Registry No. 71-36-3), hexanol (Sigma-Aldrich, 99%, CAS Registry No. 111-27-3), and decanol (Sigma-Aldrich, 99%, CAS Registry No. 112-30-1) were dried and kept under VWR Prolabo 3 Å molecular sieves, to a maximum water content of 500 ppm as analyzed by Karl Fischer coulometry. The mixtures were prepared gravimetrically using a Mettler AE 240 analytical balance (resolution of 1 × 10^–5^ g). The total uncertainty in composition is estimated to be lower than 0.001 mole fraction unit.

### Surface Tension and Density Measurements

Surface tensions were determined using the axisymmetric drop shape analysis (ADSA) method, developed by Neumann and co-workers.^46^ The measurements were performed at 298.2 ± 0.2 K by producing the drops inside a thermostatized aluminum chamber with optical windows. The temperature was measured using a calibrated platinum resistance thermometer mounted in close contact with the drop holder.^47^ Due to the hygroscopicity of the studied alcohols, the atmosphere inside the aluminum cell was kept under a low flux of dry nitrogen; the surface tension was verified to be constant as a function of time for each drop. Further details about the operation, calibration, and validation of the used experimental setup can be found in refs 48 and 49. The surface tensions determined for the pure hydrogenated alcohols studied agree within 0.2 mN m^–1^ with the reference correlations of Mulero et al.,^50^ while those obtained for the fluorinated alcohols agree within 0.1 mN m^–1^ with our previously published data.^37^ These results confirm the accuracy of the measurements performed as well as the absence of surfactant impurities in the studied compounds.

Surface tension determination with the above-mentioned method requires knowledge of the pendant drop density. The liquid densities were measured with an Anton Paar DMA 5000 vibrating-tube densimeter calibrated with water (distilled, purified with a Mili-Q 185 plus water purification system, and freshly boiled) and air between 293.15 and 333.15 K, accounting for atmospheric pressure. The calibration was checked with water over the whole range of operating temperatures, and the maximum deviation from literature values^51^ was less than 0.00002 g cm^–3^. The density of air was verified between sample measurements to ensure the cleanliness of the measurement cell. For the pure compounds, the density obtained for 1*H*,1*H*-perfluorohexanol differs less than 0.07% from a previous measurement of our group,^41^ and the densities of hexanol and decanol agree within 0.04% and 0.01%, respectively, with the reference correlations of Cibulka.^52^

### Molecular Modeling

#### Molecular Dynamics Simulations

Atomistic molecular dynamics simulations for the hexanol/1*H*,1*H*-perfluorohexanol and decanol/1*H*,1*H*-perfluorooctanol mixtures were performed using the Gromacs 5.0.7 simulation package,^53^ with the studied compounds modeled within the OPLS-AA framework.^54^ The fluorinated alcohol force fields were based on the model for 2,2,2-trifluoroethanol developed by Duffy,^55^ with the −CF_2_CH_2_OH group taken from this parametrization and the remainder perfluoroalkyl tail from Watkins and Jorgensen.^56^ The alkyl tails of the hydrogenated alcohols were modeled using the L-OPLS-AA parametrization,^57^ whereas their −CH_2_OH moiety was taken from the original OPLS-AA paper.^54^ The numerical values of the parameters used can be found in previous publications,^31,58^ and the gromacs input files used in the simulations are available in the Supporting Information.

The unlike dispersive parameters were calculated using geometric combining rules, as prescribed by the OPLS-AA force field, except the interactions between the H and F atoms of the two different components of each mixture that were adjusted to account for the peculiar volumetric and enthalpic effects that occur when highly fluorinated and hydrogenated compounds are mixed. Factors of 1.035 and 0.77 were applied to the crossed size (σ_H–F_) and energy (*ε*_H–F_) parameters, respectively, as fitted to reproduce the experimental excess volumes and excess enthalpies of alkane/perfluoroalkane mixtures in a previous work.^26^

Cubic simulation boxes with periodic boundary conditions, containing a total of 300 molecules, were generated with PACKMOL^59^ and subjected to an energy minimization to relax eventual unphysical high-energy intermolecular contacts. Then, consecutive *NpT* simulation runs were performed, using the leapfrog integrator with a 2 fs time step, constraining all bonds containing hydrogen atoms to the respective equilibrium length using the LINCS algorithm.^60^ First, a 1 ns run using the Berendsen thermostat and barostat at 600 K and 200 bar served to pre-equilibrate the systems. Then, the final boxes of the previous step were simulated at 298.15 K and 1 bar (replicating the experimental conditions), using the Nosé–Hoover thermostat^61,62^ and Parrinello–Rahman barostat,^63^ with coupling times of 0.5 and 10 ps, respectively. At least 2.5 ns of these runs was considered as the equilibration time, during which all components of the potential energy stabilized, and at least further 3 ns was used for averaging the computed properties. Beyond the cutoff distance of 1.4 nm, standard analytical corrections were applied to the energy and pressure dispersive terms and the particle-mesh Ewald method was used to account for long-range electrostatic interactions.^64,65^

### Soft-SAFT Modeling

#### Soft-SAFT Equation

The statistical association fluid theory (SAFT) stands as one of the most widely applied molecular-based equations of state for describing complex, highly nonideal fluids, particularly those involving chainlike or strongly associating molecules. Within the SAFT framework, the thermodynamic description is formulated in terms of the residual Helmholtz energy, *a* ^res^, which is expressed as the sum of the different contributions that reflect the underlying molecular structure of the fluid. In the case of associating chains, the residual Helmholtz energy can be represented as

1 $$a^{\mathrm{res}}=a^{\mathrm{ref}}+a^{\mathrm{chain}}+a^{\mathrm{assoc}}$$

where *a* ^ref^, *a* ^chain^, and *a* ^assoc^ stand for the monomer (or segment) reference fluid, the formation of the chain, and the association contribution, respectively.

In the soft-SAFT approach employed here, the reference fluid consists of Lennard-Jones (LJ) spheres, which accounts for the repulsive and attractive interactions between the monomer units.^44^ The thermodynamic properties of the reference fluid, including its free energy and the derived properties, are calculated using the accurate LJ equation of state by Johnson et al.^66^ The chain contribution, *a* ^chain^, captures the connectivity of monomers in chain molecules and is derived from the first-order Wertheim’s perturbation theory for associating spheres under the assumption of full association.^67–69^ The association contribution is also derived from Wertheim’s TPT1.^67–69^ Details of the mathematical expressions and their implementation can be found in previous publications.^44^

Using this approach, a fluid of associating chains is fully characterized in soft-SAFT by five molecular parameters: the chain length, *m*, the size of the segments making the chains, σ, the dispersive energy of interaction between segments, *ε*, and ε^HB^ and κ^HB^ related to the energy and volume of association, respectively. The chain and association terms in soft-SAFT can be directly applied to mixtures. The reference LJ term is extended to mixtures using the van der Waals one-fluid (vdW-1f) approximation, combined with the generalized Lorentz–Berthelot (LB) mixing rules:

2a $$\sigma_{ij}=\eta_{ij}\left(\frac{\sigma_{ii}+\sigma_{jj}}{2}\right)$$

2b $$\varepsilon_{ij}=\xi_{ij}\sqrt{\varepsilon_{ii}\varepsilon_{jj}}$$

where η_*ij*_ and ξ_*ij*_ are binary adjustable parameters between substances *i* and *j*. When these parameters are set equal to 1, the EoS is used in a predictive manner. The volume and energy association parameters characterizing the interactions between different association sites are calculated by mixing rules in an analogous manner

3a $$\kappa_{ij}^{\mathrm{HB}}={\left(\frac{{{(\kappa }_{ii}^{\mathrm{HB}})}^{1/3}+{{(\kappa }_{jj}^{\mathrm{HB}})}^{1/3}}{2}\right)}^{3}$$

3b $$\varepsilon_{ij}^{\mathrm{HB}}=\alpha_{ij}^{\mathrm{HB}}\sqrt{\varepsilon_{ii}^{\mathrm{HB}}\varepsilon_{jj}^{\mathrm{HB}}}$$

where α_*ij*_^HB^ is a binary adjustable parameter that can be used to correct for possible deviations from the geometric mean of the cross-association energy.

#### Soft-SAFT Equation Coupled with Density Gradient Theory

To model interfacial behavior, soft-SAFT is coupled with density gradient theory (DGT).^70,71^ The theory formulates a density functional for the local Helmholtz energy, which decomposes into homogeneous and inhomogeneous contributions. The homogeneous part is described by the Helmholtz energy density of a uniform fluid evaluated at a local density intermediate between the bulk values. In contrast, the inhomogeneous contribution depends on the local molar density, ρ­(*z*_*i*_), and its spatial derivatives, which are treated as independent variables under the assumption that the density gradient is small relative to the inverse of the intermolecular distance. Accordingly, the Helmholtz energy is expanded in a Taylor series with respect to the derivatives of the component densities along the coordinate normal to the interface and truncated at second order, yielding

4 $$a\left[\rho \right]=a_{o}\left(\rho \right)+\overset{2}{\underset{i=1}{\sum }}\overset{2}{\underset{j=1}{\sum }}\frac{1}{2}c_{ij}\nabla \rho_{i}\cdot \nabla \rho_{j}$$

where *a*_0_(ρ) is the Helmholtz energy of the homogeneous fluid at density ρ, ∇ρ_*i*_ and ∇ρ_*j*_ are the local density gradients of compounds *i* and *j*, respectively, and *c*_*ij*_ is the so-called influence parameter. This coefficient is related to the square-gradient term^72–74^ and is usually fitted to surface tension data. For mixtures, the cross-influence parameter can be obtained from those of the pure compounds using the geometric mean rule or fitted using binary adjustable parameter β_*ij*_.

5 $$c_{ij}=\beta_{ij}\sqrt{c_{ii}c_{jj}}$$

When β_*ij*_ = 1, eq 5 is used in a predictive manner from the pure component influence parameters.

The application of DGT requires the calculation of the interfacial density profiles, which have been determined following procedures described elsewhere and are not repeated here.^45^ The interfacial tension (γ) is obtained by minimizing the free energy. Further methodological details can be found in the original references.^45^

#### Soft-SAFT Molecular Models

In soft-SAFT, molecules are represented by homonuclear chains of *m* tangentially bonded LJ spheres of size σ and interaction energy *ε*. The formation of hydrogen bonds is explicitly considered in the soft-SAFT description of the alcohols by adding two associative sites to each molecule, one representing the hydrogen atom of the hydroxyl group and the other representing the lone electron pairs of the oxygen atom. Association interactions are characterized by the association volume and energy parameters, κ_*ij*_^HB^ and ε_*ij*_^HB^, respectively, and only associations between sites of different type are allowed, including those for 1*H*,1*H*-perfluorooctanol and decanol that have been obtained by extrapolation from correlations of the shorter members of the respective chemical families.^13,75–77^ The full set of parameters used is reported in Table 1.

**Table 1:** Molecular and Influence Parameters Used in the Theoretical Modeling with Soft-SAFT and DGT

substance	*m*	σ (Å)	*ε* (k_B_/K)	*k* ^HB^ (Å^3^)	*ε* ^HB^ (k_B_/K)	*c* (J m^5^ mol^–2^)
*n*-hexane	2.832	3.929	254.4	–	–	3.56 × 10^–19^
*n*-perfluorohexane	2.832	4.449	230.2	–	–	6.96 × 10^–20^
1*H*,1*H*-perfluorobutanol	2.210	4.316	239.0	2250	3450	2.34 × 10^–19^
1*H*,1*H*-perfluorohexanol	2.690	4.562	259.6	2250	3450	4.76 × 10^–19^
1*H*,1*H*-perfluorooctanol	3.180	4.718	270.9	2250	3450	8.62 × 10^–19^
butanol	2.210	3.940	269.2	2250	3450	1.54 × 10^–19^
hexanol	2.676	4.119	291.6	2250	3450	3.28 × 10^–19^
decanol	3.611	4.308	316.7	2250	3450	9.02 × 10^–19^

## Results and Discussion

### Modeling the Hexane/Perfluorohexane Mixture: Quantifying the Contribution of the Cross-Dispersive Interactions between Hydrogenated and Perfluorinated Chains

Prior to modeling the specific nonideal interactions of the alcohol/perfluoroalcohol mixtures with soft-SAFT, the dispersive size and energy binary interaction parameters (η_*ij*_ and ξ_*ij*_ in eqs 2a and 2b, respectively) were determined using the experimental VLE and excess properties of the hexane/perfluorohexane mixture.^15,78,79^ Since association (hydrogen bonding) is absent in this reference mixture, the calculated parameters characterize the purely dispersive interactions between hydrogenated and fluorinated chains, capturing the anomalies intrinsic to these interactions and isolating them from associative interactions.

The obtained parameters (η_*ij*_ = 1.012, and ξ_*ij*_ = 0.924) correspond to an increase in the cross-interaction size and a decrease in the cross-interaction energy, respectively. These corrections to the combining rules follow the same tendency as those applied to molecular simulation models^31^ and in other versions of SAFT.^80,81^ Figure 1 shows the results of the soft-SAFT calculations with the optimized parameters, the pure predictions using the combining rules (η_*ij*_ = 1, and ξ_*ij*_ = 1), and the experimental data that served as the base for the optimization process.

**Figure 1:**
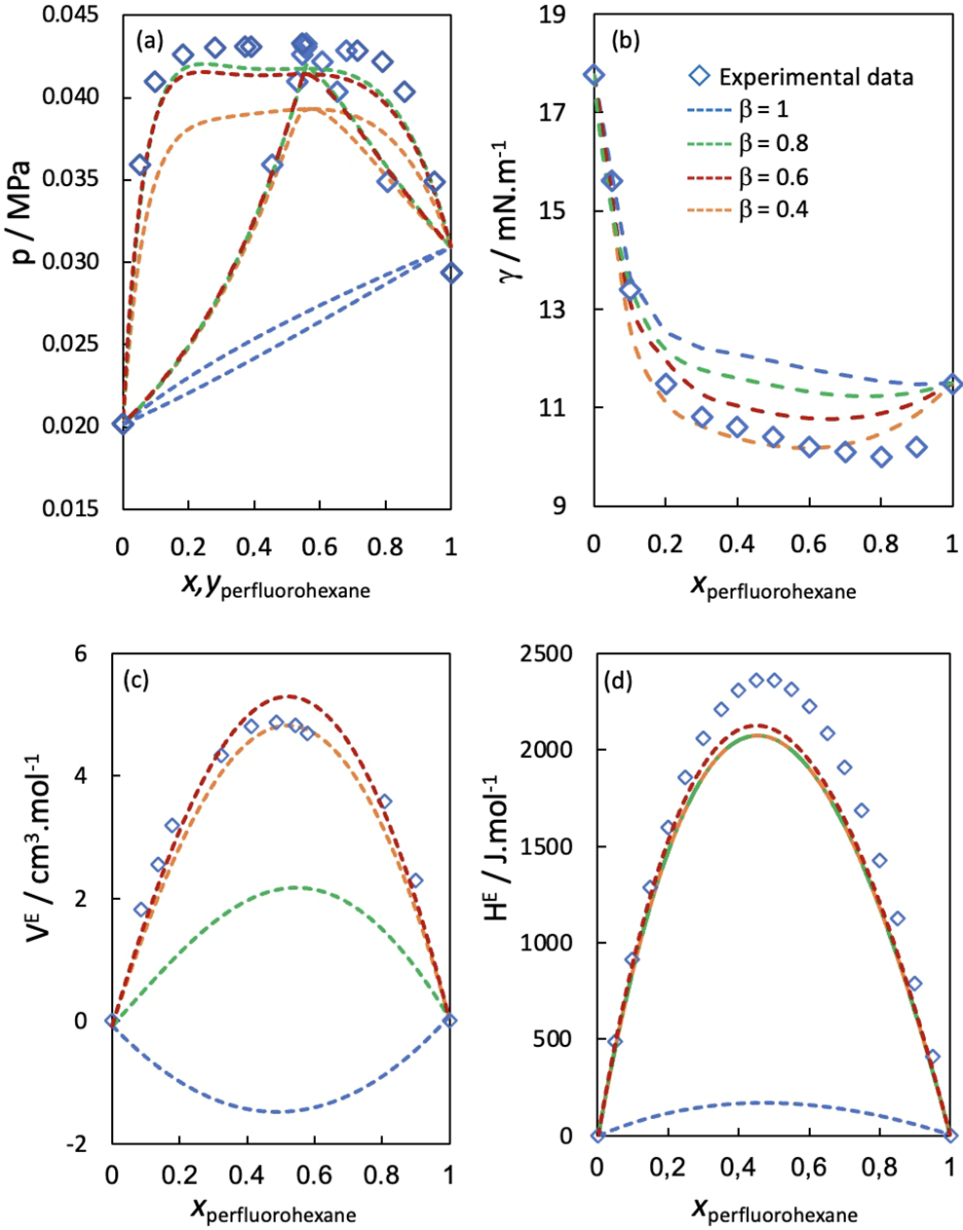
(a) Isothermal VLE phase diagram, (b) surface tension, (c) excess molar volumes, and (d) excess molar enthalpies for the hexane/perfluorohexane mixture at 298.15 K. The symbols represent experimental data,^7,15,78,79^ and the lines the soft-SAFT and soft-SAFT+DGT results. Color code in panels a, c, and d: (blue) η_*ij*_ = 1, and ξ_*ij*_ = 1; (green) η_*ij*_ = 1, and ξ_*ij*_ = 0.914; (red) η_*ij*_ = 1.012, and ξ_*ij*_ = 0.914; and (orange) η_*ij*_ = 1.012, and ξ_*ij*_ = 0.924_._ eq 5

The optimized binary interaction parameters yield a very good description of the large positive enthalpies and volumes of mixing but slightly underestimate the equilibrium pressures in the VLE diagram. The chosen ξ_*ij*_ value of 0.924 represents a compromise in capturing the maximum excess enthalpies and VLE pressures without reaching liquid–liquid separation at the studied temperature (the experimental upper critical solution temperature is ca. 295 K). In contrast, when the combining rules are used without any adjustment, the theory predicts a VLE diagram very close to ideal behavior, negative excess volumes, and only slightly positive excess enthalpies.

Finally, the interfacial behavior of the hexane/perfluorohexane mixture was modeled with soft-SAFT+DGT, using the same dispersive binary interaction parameters optimized to the bulk properties. The results are also shown in Figure 1b. As one can see, the soft-SAFT+DGT results using the combining rule for the cross-influence parameter in a predictive fashion (β_*ij*_ = 1 (blue line)) already display good agreement with the experimental data. However, to predict the aneotrope, a reduction in β*_ij_* is required, indicating that at the interface the interactions between unlike molecules are even more energetically unfavorable than in the bulk liquid.

### Modeling the Bulk Properties of Mixtures of Hydrogenated and Fluorinated Alcohols: Quantifying the Contribution of Associative Interactions

Having determined the binary dispersive parameters that account for the unfavorable interactions between the alkyl and perfluoroalkyl segments, the soft-SAFT theory was then used to predict the excess volumes and excess enthalpies of the mixtures of hydrogenated and fluorinated alcohols, to quantify the contribution of associative interactions.

The experimental excess volumes of the butanol/1*H*,1*H*-perfluorobutanol, hexanol/1*H*,1*H*-perfluorohexanol, and decanol/1*H*,1*H*-perfluorooctanol mixtures at 298.15 K were reported in previous publications^14,31^ and are compared in Figure 2a together with the soft-SAFT and molecular dynamics predictions. The excess volumes are markedly positive and increase with the chain length of the components. Nevertheless, for the hexanol/1*H*,1*H*-perfluorohexanol mixture, they are approximately half of those observed for the hexane/perfluorohexane mixture at the same temperature (Figure 1). The excess volumes obtained from the MD simulations are also shown in Figure 2a. As one can see, the simulated excess volumes are practically indistinguishable from the experimental, which can be considered a remarkable result, especially considering that the simulation models were not fitted and that only the cross-interaction parameters between fluorine and hydrogen atoms were adjusted to the properties of alkane/perfluoroalkane mixtures, as described in the previous section.

**Figure 2:**
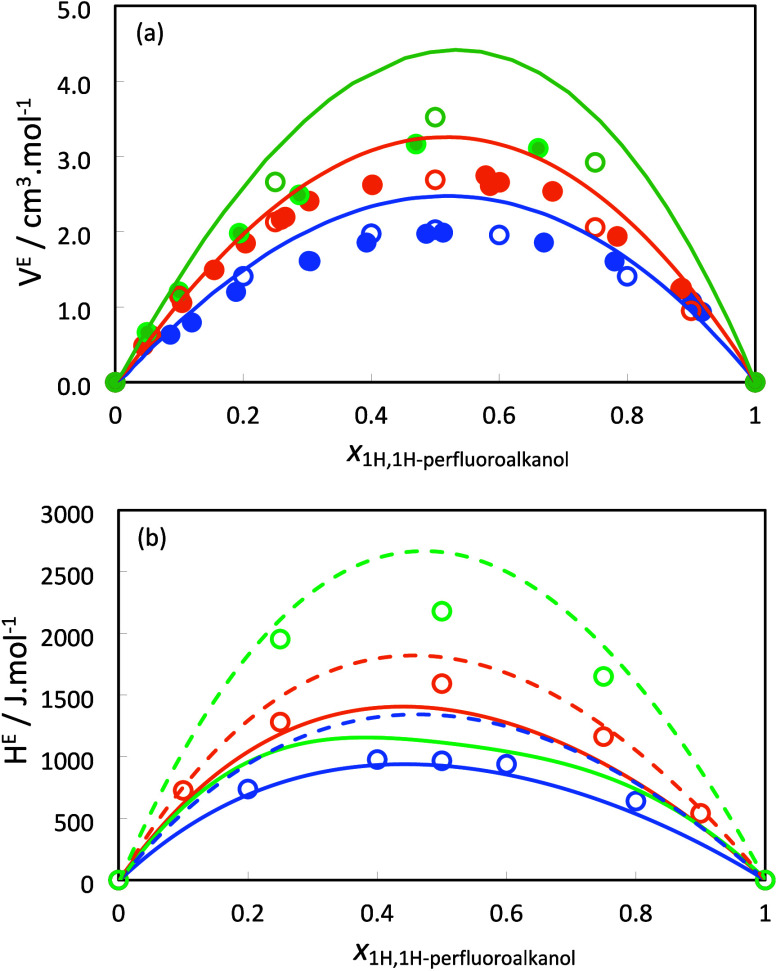
(a) Excess molar volumes and (b) excess molar enthalpies of the studied mixtures of alkanols and 1*H*,1*H*-perfluoroalkanols. Filled symbols represent the experimental results, and empty symbols the molecular dynamics simulation results; dashed lines represent the soft-SAFT calculations with η_*ij*_ = 1.012, ξ_*ij*_ = 0.924, and α_*ij*_^HB^ = 1 and solid lines the soft-SAFT calculations with η_*ij*_ = 1.012, ξ_*ij*_ = 0.924, and α_*ij*_^HB^ = 1.02 (α_*ij*_^HB^ = 1.06 for the decanol/1*H*,1*H*-perfluorooctanol mixture). Color code for the experimental results:^33^ blue for the butanol/1*H*,1*H*-perfluorobutanol mixture, orange for the hexanol/1*H*,1*H*-perfluorohexanol mixture, and green for the decanol/1*H*,1*H*-perfluorooctanol mixture.

Excess enthalpies were also obtained by MD simulation for these mixtures and are shown in Figure 2b together with the soft-SAFT calculations. Although no experimental data have been measured for these mixtures, the excellent quantitative agreement obtained for the excess volumes imparts an enhanced confidence to the excess enthalpies predicted by MD. In addition, in previous publications, we have shown that for the very small number of mixtures for which excess enthalpies have been measured experimentally, the MD simulations can accurately reproduce the experimental data. As previously discussed,^31^ the enthalpic effects that occur when hydrogenated alcohols are mixed with their fluorinated counterparts result from a balance between an endothermic contribution imparted by the unfavorable dispersive interactions of the hydrogenated and fluorinated segments, and an exothermic contribution due to the formation of more stable hydrogen bonds, namely those between the more acidic fluorinated alcohol, acting as a donor, to the more basic hydrogenated one. The simulation results in Figure 2b follow the same trend. The excess enthalpies become more endothermic as the chain length of the components increases, due to the progressive predominance of the dispersive component of the mixture energy, which is dominated by the unfavorable hydrogenated–fluorinated interactions.

The soft-SAFT predictions for the bulk excess properties of the studied alcohol mixtures are also shown in Figure 2. As shown in Figure 2b, soft-SAFT overestimates the excess enthalpies if only the dispersive cross-parameters previously obtained (η_*ij*_ = 1.012, and ξ_*ij*_ = 0.924 (eqs 2a and 2b)) are used, uncovering the need to account for the enhanced cross-hydrogen bond between hydrogenated and fluorinated alcohols. This is achieved by slightly increasing the α_*ij*_^HB^ parameter in the combining rule for the crossed association energy (eq 3b) from 1 to 1.02. For the decanol/1*H*,1*H*-perfluorooctanol mixture, a slightly larger α_*ij*_^HB^ value of 1.06 was needed, as soft-SAFT predicts liquid–liquid phase separation for lower values of α_*ij*_^HB^ at the studied temperature. Hence, all further calculations were made with the binary interaction parameters η_*ij*_ = 1.012 and ξ_*ij*_ = 0.924, while α_*ij*_^HB^ = 1.02 was used for butanol/1*H*,1*H*-perfluorobutanol and hexanol/1*H*,1*H*-perfluorohexanol mixtures and α_*ij*_^HB^ = 1.06 for the decanol/1*H*,1*H*-perfluorooctanol mixture.

The excess volumes calculated with soft-SAFT are also depicted in Figure 2a. As one can see, the theory reproduces both the order of magnitude and the trend between the systems. The observed overestimations can be explained, at least in part, by the homonuclear nature of the adopted soft-SAFT molecular model. The studied alcohols are not fully fluorinated molecules but have a −CH_2_OH group. However, the cross-size correction parameter, η_*ij*_, is applied to the interactions between all groups in the fluorinated molecule and all groups in the hydrogenated molecule. This corresponds to a small overcorrection that enhances the volumetric effect of the unfavorable interaction between the two types of chains. It should be remembered that the calculated excess volumes are fully determined by cross-size correction η_*ij*_, being practically independent of the value of energetic parameters ξ_*ij*_ and α_*ij*_^HB^ (see Figure 1).

Finally, soft-SAFT was used to calculate the liquid–vapor equilibrium phase diagrams of the three mixtures that, to the best of our knowledge, have not been measured experimentally. The results, which are pure predictions, are presented as Supporting Information.

### Interfacial Behavior

The liquid–vapor surface tension of the three mixtures of medium/long chain hydrogenated and perfluorinated alcohols was measured at 298.2 K, in the full composition range. The results are reported in Table 2 and are represented in Figure 3. The decanol/1*H*,1*H*-perfluorooctanol mixture was only measured up to the limit of solubility of 1*H*,1*H*-perfluorooctanol, which is a solid at the working temperature. To help the discussion and to allow the theoretical treatment of this system with soft-SAFT+DGT, we have estimated the hypothetical surface tension of liquid (supercooled) 1*H*,1*H*-perfluorooctanol at 298.2 K by extrapolating the tendency of the three immediately shorter elements of the chemical family previously measured in our group, 1*H*,1*H*-perfluoropentanol, 1*H*,1*H*-perfluorohexanol, and 1*H*,1*H*-perfluoroheptanol.^37^

**Table 2:** Experimental Surface Tensions of Butanol/1*H*,1*H*-Perfluorobutanol, Hexanol/1*H*,1*H*-Perfluorohexanol, and Decanol/1*H*,1*H*-Perfluorooctanol Mixtures at 298.2 K

butanol and 1*H*,1*H*-perfluorobutanol		hexanol and 1*H*,1*H*-perfluorohexanol		decanol and 1*H*,1*H*-perfluorooctanol	
*x*_perfluorobutanol_	γ (mN m^–1^)	*x*_perfluorohexanol_	γ (mN m^–1^)	*x*_perfluorooctanol_	γ (mN m^–1^)
0	23.93	0	25.80	0	27.98
0.086	21.29	0.026	23.76	0.049	24.35
0.120	20.72	0.044	22.76	0.099	22.79
0.190	19.87	0.056	22.41	0.194	21.18
0.302	18.92	0.075	21.96	0.288	20.20
0.305	19.09	0.104	21.41	0.410	19.67
0.390	18.30	0.155	20.93	0.506	18.81
0.393	18.19	0.204	20.21	0.599	18.58
0.486	17.84	0.259	19.72	1	17.6^a^
0.512	17.40	0.303	19.16		
0.624	17.16	0.402	18.69		
0.670	17.14	0.491	18.36		
0.780	16.91	0.586	17.57		
0.850	16.60	0.601	17.97		
0.900	16.52	0.683	17.44		
0.920	16.53	0.785	17.21		
1	16.81	0.883	17.30		
		0.885	16.85		
		0.950	17.16		
		1	16.97		

a Estimated value, extrapolated from the shorter members of the fluorinated alcohols series (see the text for details).

**Figure 3:**
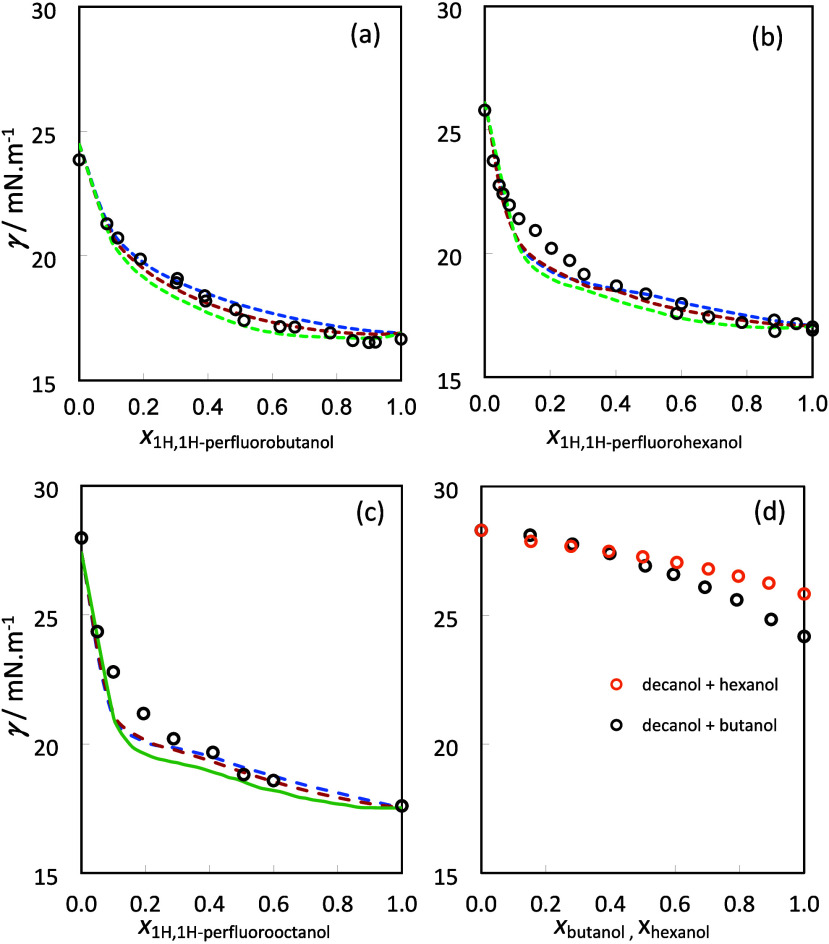
Surface tension of (a) butanol/1*H*,1*H*-perfluorobutanol, (b) hexanol/1*H*,1*H*-perfluorohexanol, and (c) decanol/1*H*,1*H*-perfluorooctanol mixtures at 298.2 K. Empty symbols represent the new experimental data, and lines the soft-SAFT+DGT predictions with β_*ij*_ = 1 (blue), β_*ij*_ = 0.9 (red), and β_*ij*_ = 0.8 (green). (d) Decanol/butanol and decanol/hexanol experimental data from ref 82.

The three mixtures show a very similar interfacial behavior. For compositions rich in the hydrogenated component, the surface tension rapidly decreases with the addition of the fluorinated alcohol, confirming its strong tendency to adsorb. At higher concentrations of the fluorinated component, the surface tension decreases at a lower rate. In the region close to the pure fluorinated component, the butanol/1*H*,1*H*-perfluorobutanol mixture displays a shallow negative aneotrope. The hexanol/1*H*,1*H*-perfluorohexanol mixture also seems to display an even shallower aneotrope close to that of pure 1*H*,1*H*-perfluorohexanol, although within experimental uncertainty. For the decanol/1*H*,1*H*-perfluorooctanol mixture, the solubility limit precludes the eventual observation of this phenomenon. These results agree with the trend previously observed for mixtures of 2,2,2-trifluoroethanol (TFE) with the hydrogenated alcohols, ethanol, propanol, and butanol,^13^ which display well-defined negative aneotropes that become less pronounced and progressively deviate toward compositions closer to the fluorinated component, as the chain length of the hydrogenated alcohol increases.

The three isotherms display very large deviations from the weighted average surface tension of the pure components. Although the concept of “ideal interfacial behavior” is not defined, this deviation is often called excess surface tension (γ^E^ = γ – (*x*_1_γ_1_° + *x*_2_γ_2_°)) and is a useful quantity to compare surface tension curves of different mixtures, involving components of different absolute surface tension. It can be interpreted as a deviation to an “operational” surface ideality.

The excess surface tensions for the studied mixtures are shown in Figure S2. As one can see, the γ^E^ for all three mixtures is large and negative and becomes more negative with an increase in chain length, reaching −5 mN m^–1^ for the decanol/1*H*,1*H*-perfluorooctanol mixture, a value very close to the excess surface tension of the hexane/perfluorohexane mixture, also included in Figure S2 for comparison. It can also be observed that the shape of the curves is very similar for all mixtures, displaying a minimum at approximately 0.2 mole fraction of the perfluorinated component.

Comparing the surface tension isotherms for the three alcohol mixtures with that of the hexane/perfluorohexane mixture shows that introducing an associative OH group considerably reduces the magnitude of the aneotrope. Increasing the chain length of both alcohols, and thus the contribution of the unfavorable dispersion forces, further reduces the magnitude of the aneotrope and deviates the aneotropic composition toward the perfluorinated component until it eventually disappears.

Comparing the same three isotherms with those of mixtures of hydrogenated alcohols uncovers the contribution to the interfacial behavior of mixing hydrogenated and perfluorinated chains. With that in mind, Figure 3 also includes the surface tension isotherms from the literature for two mixtures of hydrogenated alcohols of comparable chains lengths, decanol with butanol and decanol with hexanol.^82^ As one can see, the interfacial behavior of these mixtures is very different from that observed for the systems reported in this work. The excess surface tensions are now small and positive, not exceeding 0.75 mN m^–1^ (see Figure S2). This comparison clearly demonstrates that the interfacial anomalies displayed by the mixtures of hydrogenated and perfluorinated alcohols are a consequence of the mutual phobicity between the hydrogenated and fluorinated chains, further influenced by the orientational constraints induced by the interface.

The soft-SAFT+DGT predictions for the surface tension of the studied mixtures calculated using the same molecular models and the binary interaction parameters optimized to the bulk properties are also depicted in Figure 3. As one can see, the soft-SAFT+DGT calculations using the combining rule for the cross-influence parameter (eq 5) in a predictive fashion (β_*ij*_ = 1) already show very good agreement with experiments. Despite not showing the presence of the aneotrope, these pure predictions reproduce the general shape of the surface tension curves, showing the enhanced adsorption of the fluorinated alcohol at low concentrations and the further slower evolution toward the surface tension of this pure component. An even better agreement, already displaying an aneotrope in all mixtures, can be obtained reducing binary influence parameter β_*ij*_ to 0.8, the value obtained in a previous work for mixtures of 2,2,2-trifluoroethanol with shorter hydrogenated alcohols, ethanol, propanol, and butanol.^13^ This reduction of the cross-influence parameter reflects the fact that at the interface the interactions between unlike molecules are even more energetically unfavorable than in the bulk liquid. The molecular orientation constraints induced by the interface probably reduce the likelihood of forming the energetically favorable cross hydrogen bonds, enhancing the effect of the unfavorable fluorinated and hydrogenated dispersive interactions.

Taking advantage of the molecular nature of the soft-SAFT equation, the previous results were complemented by calculations of the relative adsorptions and density profiles. Relative adsorptions Γ_12_ of the fluorinated alcohols (1) relative to the hydrogenated alcohols (2) in the full composition range were calculated using eq 6 and are shown in Figure 4.

6 $$\Gamma_{12}=-\left(\rho_{1}^{L}-\rho_{1}^{V}\right)\int_{-\infty }^{+\infty }\left(\frac{\rho_{2}\left(z\right)-\rho_{2}^{L}}{\rho_{2}^{L}-\rho_{2}^{V}}-\frac{\rho_{1}\left(z\right)-\rho_{1}^{L}}{\rho_{1}^{L}-\rho_{1}^{V}}\right)$$

where ρ_*i*_^L^ and ρ_*i*_^V^ are the densities of species *i* in the liquid and gaseous phases, respectively, and ρ_*i*_(*z*) is the density of species *i* at a flat liquid–vapor interface *z*.^12^

**Figure 4:**
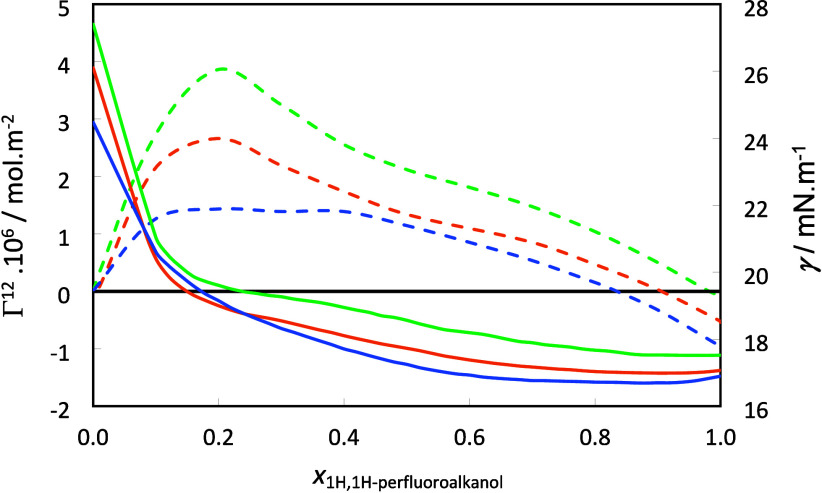
Surface tension, γ (solid line), and relative adsorption of the fluorinated alcohol, Γ_12_ (dashed line), as a function of the mole fraction of the fluorinated alcohol for the studied mixtures. Color code: blue for the butanol/1*H*,1*H*-perfluorobutanol mixture, orange for the hexanol/1*H*,1*H*-perfluorohexanol mixture, and green for the decanol/1*H*,1*H*-perfluorooctanol mixture.

As expected, all of the systems show positive adsorption of the fluorinated alcohol at low compositions, reaching a maximum at around *x*_fluorinated_ = 0.25 and remaining positive up to the aneotropic composition, where no adsorption is observed (Γ_12_ = 0). As the composition shifts from the aneotrope toward the pure fluorinated component, the relative adsorption becomes progressively more negative, reflecting the interfacial adsorption of the hydrogenated alcohol.

This trend is illustrated in Figure 5, which shows the interfacial density profiles obtained with soft-SAFT+DGT for both components in each mixture, at three representative compositions: near the extremities of the diagram (*x*_fluorinated_ = 0.01, and *x*_fluorinated_ = 0.99) and at the aneotrope composition calculated by the theory (*x*_fluorinated_ = 0.82 for the butanol/1*H*,1*H*-perfluorobutanol mixture, *x*_fluorinated_ = 0.90 for the hexanol/1*H*,1*H*-perfluorohexanol mixture, and *x*_fluorinated_ = 0.98 for the decanol/1*H*,1*H*-perfluorooctanol mixture. All of the interfaces present a very large enrichment in the fluorinated alcohol at the lowest composition, reflecting the high surfactant activity of these components in the studied systems. On the opposite side of the diagram, all of the hydrogenated alcohols also show interfacial adsorption, although in a less evident fashion. This is especially true in the case of the hexanol/1*H*,1*H*-perfluorohexanol and decanol/1*H*,1*H*-perfluorodecanol systems, where the calculated aneotropes are quite shallow. At the aneotrope, no adsorption is observed, showing that the composition at the interface is equal to that of the bulk solution.

**Figure 5:**
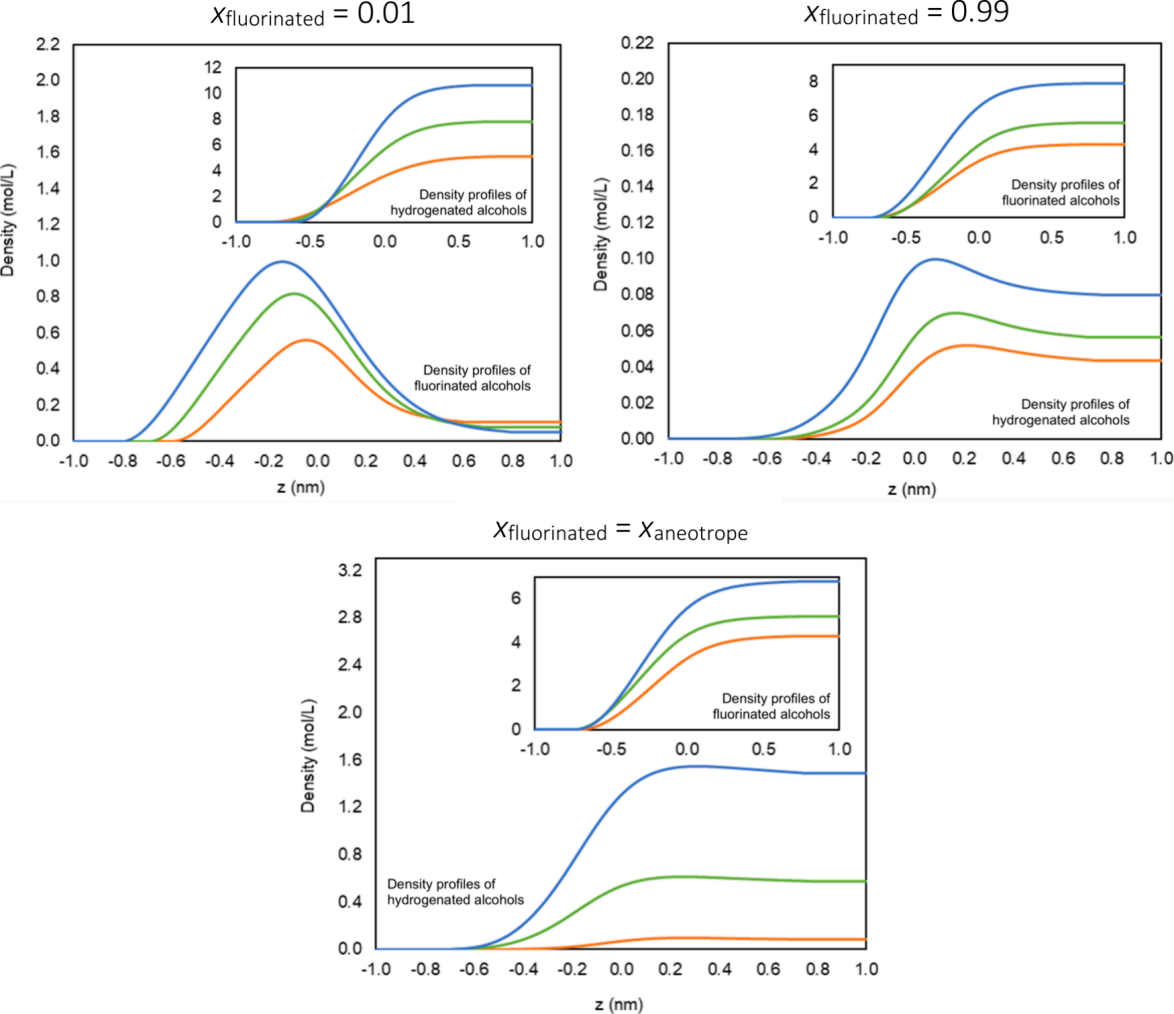
Interfacial density profiles obtained from the soft-SAFT+DGT calculations for both components at three compositions: top left, *x*_fluorinated_ = 0.01; top right, *x*_fluorinated_ = 0.99; bottom, *x*_fluorinated_ = *x*_aneotrope_. Color code: blue for the butanol/1*H*,1*H*-perfluorobutanol mixture, green for the hexanol/1*H*,1*H*-perfluorohexanol mixture, and orange for the decanol/1*H*,1*H*-perfluorooctanol mixture.

## Conclusions

This work advances the understanding of the origin of aneotropy and related interfacial anomalies by combining theoretical calculations using the soft-SAFT and soft-SAFT-DGT theories, atomistic MD simulations, and new experimental data. A systematic strategy was followed to isolate and quantify the contributions of different molecular interactions that determine the bulk and interfacial behavior of highly nonideal mixtures.

The bulk properties (VLE and large positive *V* ^E^ and *H* ^E^) of hexane and perfluorohexane were first modeled with soft-SAFT. Since this mixture is governed exclusively by dispersion forces, responsible for the mutual phobicity between hydrogenated and perfluorinated chains, this permitted us to see the influence of the cross-dispersive interactions and to parametrize them for further use with the theoretical model. Soft-SAFT-DGT was then used to model the interfacial properties of the mixture, predicting the negative aneotrope and the large negative “excess surface tension”. This reveals that the weak interactions between unlike molecules, responsible for the large deviations to ideality in the bulk liquid, are even more unfavorable at the interface, leading to the large surface anomalies.

The effect of introducing an associative OH group in molecules of equivalent chain length was addressed next, by studying three mixtures of hydrogenated and fluorinated alcohols. New experimental surface tension isotherms were reported for the butanol/1*H*,1*H*-perfluorobutanol, hexanol/1*H*,1*H*-perfluorohexanol, and decanol/1*H*,1*H*-perfluorooctanol systems. The mixtures display very large negative “excess surface tensions” and in some cases shallow negative aneotropes, shallower than that displayed by the hexane/perfluorohexane mixture. The results corroborate and extend to longer chains those of a previous work involving short chain alcohols, completing the understanding of the influence of competing molecular forces on the macroscopic behavior of these highly nonideal mixtures.

The bulk properties of the mixtures were modeled with MD simulations and soft-SAFT to parametrize the cross-associative interactions, separately from the dispersive interactions previously treated. The interfacial behavior of the three mixtures of hydrogenated and fluorinated alcohols was predicted with soft-SAFT-DGT. The theoretical predictions reproduce the experimental data and show that the observed surface anomalies can be accounted for by enhancing the contribution of the weak cross-dispersive forces between unlike molecules.

Overall, this work provides a deeper molecular-level interpretation of the unusual interfacial anomalies observed in highly nonideal mixtures, contributing to the rational understanding and future design of such systems for a range of technological applications.

## Supplementary Material


