# Supplementary material for: Molecular Origin of Aneotropy and Related Surface Tension Anomalies in Hydrogenated and Fluorinated Alcohol Mixtures: New Experimental Data and Theoretical Molecular Modeling
Source: Langmuir. 2026 Apr 9;42(15):10485–95. doi: 10.1021/acs.langmuir.6c00218 (PMC13104174; doi:10.1021/acs.langmuir.6c00218)
Supplement: Supplementary file 1 [file la6c00218_si_001.pdf]

Supporting Information

Molecular Origin of Aneotropy and Related Surface Tension Anomalies in Hydrogenated + Fluorinated Alcohol Mixtures: new Experimental Data and Theoretical Molecular Modelling

João Duarte[1,2], Diogo Machacaz[1], Tiago M. Eusébio[1], Teresa Pires[1], Pedro Morgado[1], Lourdes F. Vega[2,3*] and Eduardo J. M. Filipe[1*]

[1]Centro de Química Estrutural, Institute of Molecular Sciences, Instituto Superior Técnico, Universidade de Lisboa, 1049-001 Lisboa, Portugal

[2]Research and Innovation Center on CO_2 and Hydrogen (RICH Center), Khalifa University of Science and Technology, PO Box 127788, Abu Dhabi, United Arab Emirates

[3]Department of Chemical and Petroleum Engineering, Khalifa University of Science and Technology, PO Box 127788, Abu Dhabi, United Arab Emirates

Number of pages: 3

Number of figures: 2

Table of Contents

Figure S1. VLE phase diagrams for mixtures of fluorinated and hydrogenated alcohols at 298.2K.

Figure S2. Excess surface tension at 298.2K of mixtures: (●) (butanol + 1H,1H-perfluorobutanol), (●) (hexanol + 1H,1H-perfluorohexanol), (●) (decanol + 1H,1H-perfluorooctanol), (●) (hexane + perfluorohexane), (●) (decanol + butanol) and (●) (decanol + hexanol).

Figure S1. VLE phase diagrams for mixtures of fluorinated and hydrogenated alcohols at 298.2K.

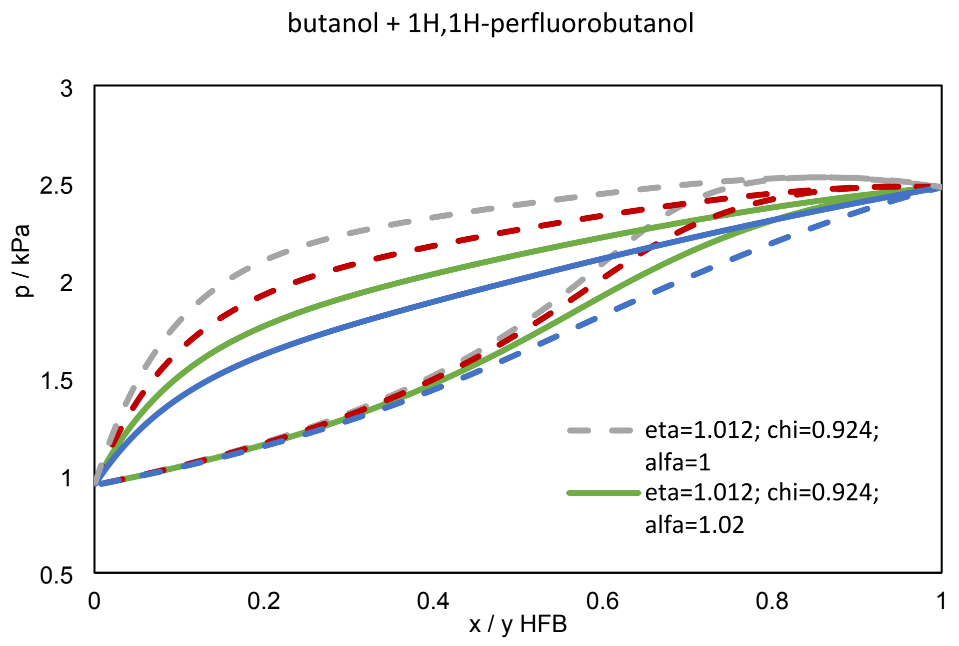

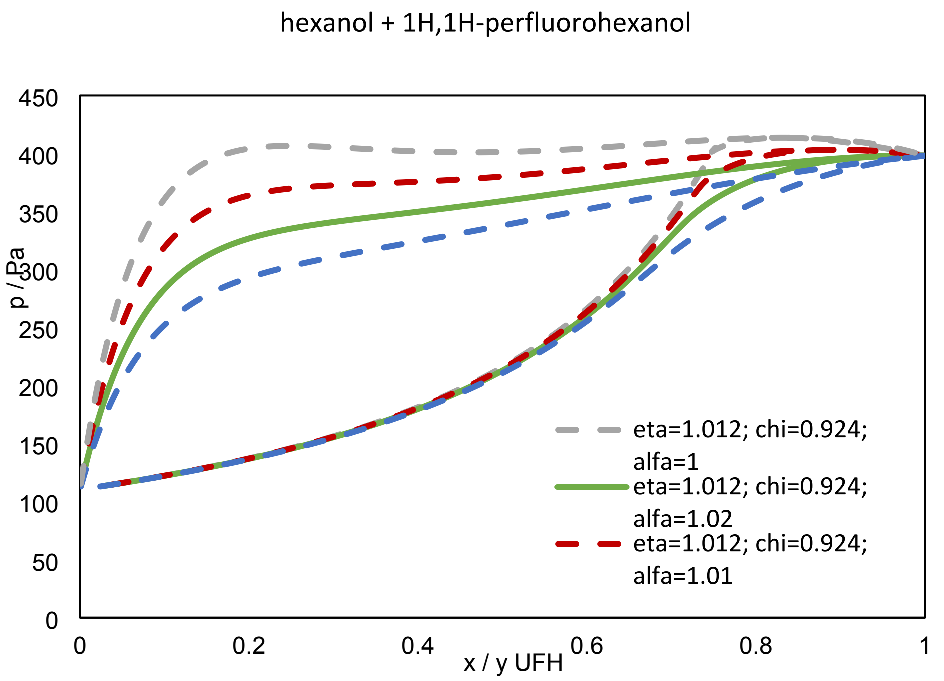

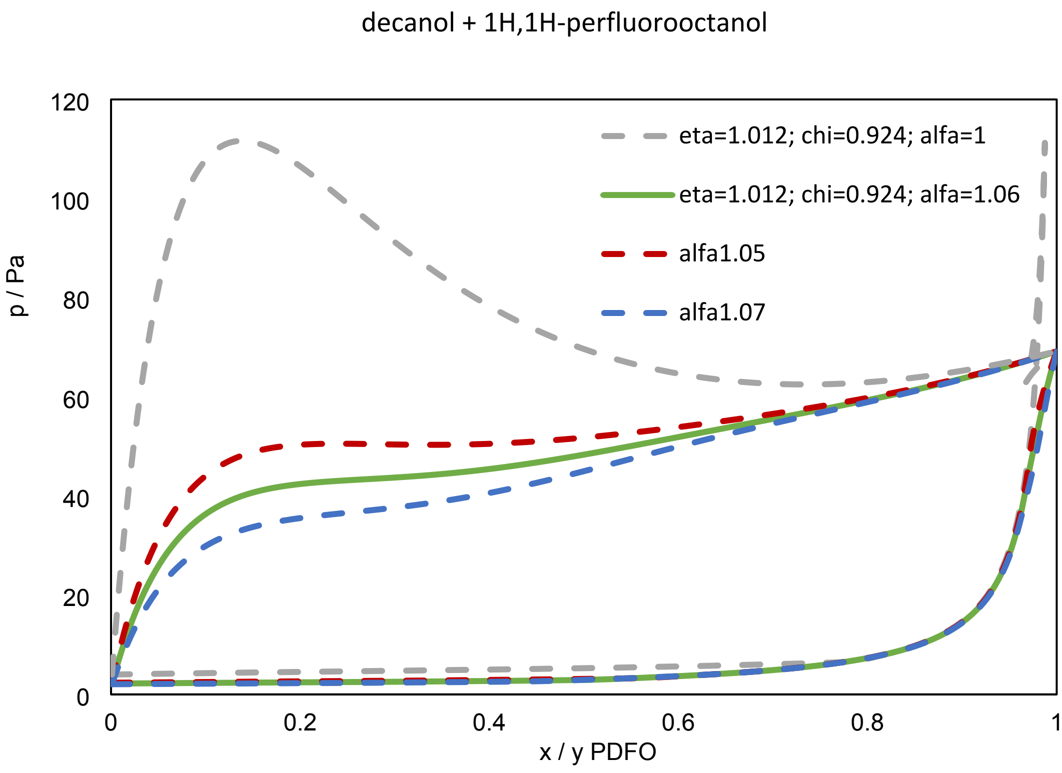

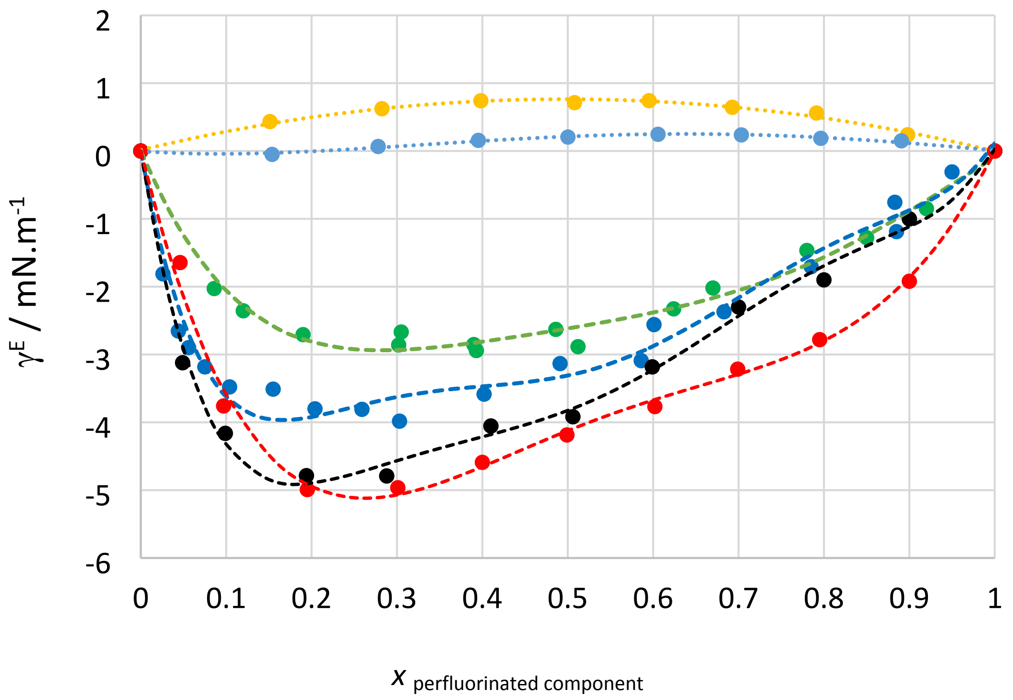

Figure S2. Excess surface tension at 298.2K of mixtures: (●) (butanol + 1H,1H-perfluorobutanol), (●) (hexanol + 1H,1H-perfluorohexanol), (●) (decanol + 1H,1H-perfluorooctanol), (●) (hexane + perfluorohexane), (●) (decanol + butanol) and (●) (decanol + hexanol).
